# Towards Improvements in the Estimation of the Coalescent: Implications for the Most Effective Use of Y Chromosome Short Tandem Repeat Mutation Rates

**DOI:** 10.1371/journal.pone.0048638

**Published:** 2012-10-31

**Authors:** Steven C. Bird

**Affiliations:** Department of Biology, Texas State University, San Marcos, Texas, United States of America; University of Massachusetts, United States of America

## Abstract

Over the past two decades, many short tandem repeat (STR) microsatellite loci on the human Y chromosome have been identified together with mutation rate estimates for the individual loci. These have been used to estimate the coalescent age, or the time to the most recent common ancestor (TMRCA) expressed in generations, in conjunction with the average square difference measure (ASD), an unbiased point estimator of TMRCA based upon the average within-locus allele variance between haplotypes. The ASD estimator, in turn, depends on accurate mutation rate estimates to be able to produce good approximations of the coalescent age of a sample. Here, a comparison is made between three published sets of per locus mutation rate estimates as they are applied to the calculation of the coalescent age for real and simulated population samples. A novel evaluation method is developed for estimating the degree of conformity of any Y chromosome STR locus of interest to the strict stepwise mutation model and specific recommendations are made regarding the suitability of thirty-two commonly used Y-STR loci for the purpose of estimating the coalescent. The use of the geometric mean for averaging ASD and 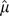 across loci is shown to improve the consistency of the resulting estimates, with decreased sensitivity to outliers and to the number of STR loci compared or the particular set of mutation rates selected.

## Introduction

Two types of genetic markers found on the human Y chromosome are used extensively in the fields of forensics, genetic anthropology, population genetics and for identification of kinship among individuals; the first is the single nucleotide polymorphism (SNP) and the second is the short tandem repeat (STR), also called the microsatellite [1]. SNPs consist of random single point mutations within an individual genome that are passed down to the descendants of the first individual to acquire the specific SNP. SNPs are generally assumed to mutate approximately according to the Infinite Alleles Model (IAM), where each mutation event is unique and independent of all other SNPs [2]. The IAM was determined to be a reasonably accurate model of SNP mutation for either the Y chromosome or for mitochondrial DNA unless a very small effective (breeding) population size (N_e_<200) was present [2].

The other type of genetic marker, the STR, consists of short, repeated segments of non-coding DNA (also called microsatellites) that increase or decrease in length by one or more sets of repeats (ex. GATA/GATA/GATA to GATA/GATA/GATA/GATA, etc.) which are typically characterized by the number of repeats that appear within a given chromosomal segment site [3], [4]. These loci are classified according to the length of the relevant microsatellite repeat sequence, i.e., dinucleotides (AT/AT/AT etc.) trinucleotides (AAT/AAT/AAT etc.), tetranucleotides (GGAT/GGAT/GGAT etc.), pentanucleotides, and hexanucleotides. STRs are assumed, generally, to mutate according to a different model, the Stepwise Mutation Model (SMM) [5], [6]. A full mathematical treatment of both mutation models, as they applied to the Y chromosome, was made by [2].

Human Y-SNPs have a low mutation rate, approximately 2.0E-8 [7], but Y-STRs mutate much more quickly, on the order of 1.0E-4 to 1.0E-2, with each STR having its own mutation rate [3], [4]. Because they are treated as unique evolutionary polymorphisms (UEPs), SNPs may be used to differentiate divisions (clades, also called haplogroups) of the overall human phylogenetic tree, particularly those SNPs associated with the two uniparentally inherited chromosomes, the Y chromosome (passed from the father only to his male offspring) [1] and the mitochondrial DNA chromosome (passed from the mother to all of her offspring, male or female) [8].

Differences between human mitochondrial DNA chromosomes are defined solely by UEPs (SNPs) while the Y chromosome features both SNPs and STRs, allowing a more detailed resolution of the genetic history of a given individual or population of males [1]. Genetic profiles formed through the aggregation of various allele repeat values (e.g, 13-24-13-10-11-12-13-30 for eight STR loci) at multiple Y-STR locus sites across an individual’s Y chromosome are called haplotypes and are in 100% linkage disequilibrium with each other, due to non-recombination of the SRY region during meiosis; these allele values therefore are subject to change only when mutation occurs at a given individual STR locus [1]. The combination of SNPs and STRs provides a powerful tool for delineating male population substructure; male individuals who share the same Y-SNP also must share a common male lineal ancestor at the point of the SNP’s first appearance, while STRs can be used to estimate the number of generations that have passed since the haplogroup’s most recent common ancestor (MRCA) lived [1], [9].

For the Y chromosome, coalescence is the process by which two or more haploid male gene genealogies, drawn as a sample from a current population (generation 0,) converge after *t* generations at the MRCA [10]. Y-STR comparisons have been used for over a decade as a means of estimating the coalescent age parameter (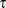) in generations for a sample, e.g., [11], by measuring the average genetic distance between haplotypes. Two very similar measures of genetic distance for STR allele data, 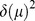 and 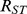
were published almost simultaneously in 1995, with each measure referred to as “average squared difference” and “average squared distance” respectively [12], [13]. The 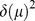
(henceforth ASD) method calculated the ratio of the observed within-locus allele variance, averaged across all sampled loci 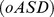 and divided by the mean of individual 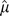 across all sampled loci 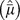, thus 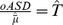, with the parameter 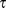 estimated in generations to the common ancestor (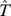). Unlike 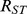, ASD was determined to be independent of population size when populations were in mutation-drift equilibrium [12].

Under the idealized Strict Stepwise Mutation Model (S-SMM) there is a linear relationship expected between 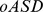 and 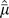; any deviations from linearity are due only to stochasticity in the allele mutation process, genetic drift within the sampled population or sampling error [14]. ASD’s linearity was lost only within a population undergoing size change and only until its variance assumed a new equilibrium value, but this loss of linearity was restricted only to the interval when the population was out of mutation-drift equilibrium [12]. It was reported subsequently by the same authors that there was no evidence to support the hypothesis that Y microsatellites were out of mutation-drift equilibrium [15]. ASD also has been shown empirically to be a reliable molecular clock and an unbiased estimator of TMRCA, with a linearity that extended to more than two million years before present in humans and a high correlation coefficient (R^2^ = 0.97) with genetic sequence divergence measures based on nucleotide substitutions [16]. Another attractive property of ASD, as a model-free means of calculating an unbiased point estimate for 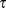, is its independence from the shape of a population’s genealogy, size or growth rate [17].

Some problems remain with the accuracy of 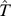, however. In particular, the selection of appropriate mutation rates for use when calculating 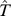 is an ongoing topic of controversy [18], [19], [20]. An accurate estimate of the coalescent is strongly dependent upon the quality of the estimates of 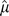 [20]. There has been no systematic comparison made yet, however, of different sets of published Y STR mutation rates with respect to their ability to produce an accurate estimate of 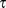. Herein, I examined the effects of these differences in 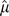 on the calculation of 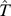 by comparing estimates generated from these three published sets of Y-STR mutation rates while using the average square difference (ASD) approach to calculate the coalescent.

Almost 200 STR loci and their individually estimated mutation rates (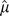) have been identified in the non-recombining (NRY) region of the human Y chromosome [3], [4]. A recent study has estimated 186 Y-STR mutation rates using a Bayesian posterior distribution analysis, detecting 82 loci (44%) in the range of 1.0×10^−4^, 91 loci (48.9%) with mutation rates in the range of 1.0×10^−3^ and 13 (6.9%) in the 1.0×10^−2^ range, covering two orders or magnitude [3]. Another made estimates for 110 STR loci based on a logistic regression model and ranged from 3.6×10^−4^ per generation to 9.6×10^−3^ [4]. A third set of estimates maintained by the Y Chromosome Haplotype Reference Database (www.YHRD.org), also is used widely. Two types of Y-STRs are used typically in genetic research and applications: (1) single-site STRs (ssSTRs) containing only one uninterrupted variable stretch of repeats and (2) multi-site STRs (msSTRs) where more than one site on the chromosome binds with the primer used to determine the number of allele repeats present at the STR of interest. Single-site STRs have a more linear relationship between mutation rates and accumulated variance than msSTRs [16], a finding with direct relevance to the calculation of the coalescent.

The estimation and use of a correct mutation rate is problematic with regard to msSTRs, such as DYS 385a/b or DYS464a/b/c/d, because it is not possible under standard genotyping protocols (without direct sequencing) to distinguish which of the duplicated sites has mutated [21], [22]. If independent mutational mechanisms occur with each part of a multi-part STR [3], then without knowledge of the individual per-copy mutation rates or the ability (generally) to distinguish one copy of the msSTR from another, the ascertainment problem will be compounded by the potential for application of an incorrect mutation rate estimate to the wrong locus.

A different type of problem arises with the multi-part locus DYS389. Rather than being a duplicated STR locus, DYS389 is a complex consisting of one polymorphic region, DYS389I, contained entirely within another, larger polymorphic region, DYS389II, both of which are amplified by the primer [21]. Because DYS389I is contained entirely within DYS389II, the allele value of DYS389II is partly dependent on the value of DYS389I. Rather than having a multipart locus with two identical or nearly identical parts that cannot be distinguished easily, the DYS389 locus has one independent and one dependent part. A mutation in DYS389I will change the allele value of DYS389II, but the reverse is not true; a mutation occurring outside of the DYS389I portion will not change the allele value of DYS389I. Thus, this locus will suffer from collinearity – two apparently independent variables that are in fact different expressions of the same variable – at least part of the time if both DYS389I and DYS389II are used in the calculation of 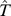.

Another potential source of bias in the estimation of 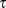
may be introduced by the use of one or more ssSTR loci that do not adhere closely to the S-SMM. For example, DYS392 and DYS438 were found to be bimodal in their distributions of allele repeat values, with certain pairs of allele values for both loci also having high linkage disequilibrium measures, D’ = 0.70 for {DYS392 = 11,DYS438 = 10} and D’ = 0.72 for {DYS392 = 13,DYS438 = 12}; both pairings were associated with each other strongly in real populations [23]. The authors attributed these unusual allele distributions to ancient demographic events rather than departures from the stepwise mutation model, but the consequences for the accurate calculation of the coalescent were not explored in detail.

The problem of bimodal allele value distributions also was described by [9], with the finding that the bimodal distribution of DYS392 differed substantially between two haplogroups as defined by “Unique Mutation Events” (UMEs, identical to UEPs or SNPs). The author of [9] attributed this phenomenon to a “bottleneck” effect, with UMEs, defining newly emerged Y haplogroups as “bottlenecks.” (Note that the use of the term bottleneck in [9] differs from the more conventional definition used within population biology, i.e., a sharp decline in breeding individuals within a population, leading to greatly reduced genetic diversity among the descendants of the surviving individuals.) [9] noted that the net result of this effect was to cause an apparent breakdown of linkage disequilibrium, resulting in differences in genetic variation between Y haplogroups for DYS392. As part of this study, statistical analyses of STR data sets with and without these loci present (shown below) have identified significant biasing effects from DYS392 and DYS438, when used together, on the estimation of 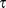.

The Strict Stepwise Mutation Model (S-SMM) first described the process of genetic mutation for STRs [5], [6]. Alleles increased or decreased by one set of repeats when copied during meiosis according to an expected rate of mutation, 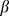, equivalent to 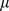 (the per-locus mutation rate) in other studies [12], [15]. The expected change in one generation was expressed as:
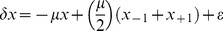
where 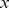 was for a starting allele repeat count in a population, 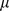 was for the per-locus mutation rate, 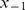 and *x*_+1_ denoted the repeat counts adjacent to *x*, either 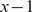 or 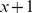 repeats, respectively, and 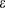 was for change due to random sampling of gametes [6].

A modification of the S-SMM, called the Generalized Stepwise Mutation Model (G-SMM) was developed by [23]. In the process of extending the G-SMM, several important characteristics of the S-SMM’s distribution were described by [25]. If 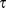 was large, then the Poisson probability distribution of the idealized S-SMM had a kurtosis (*Κ*) of 3.0. If *K* was large, however, then the distribution had a heavy tail and any estimate of 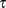
using STRs was difficult. For microsatellites, kurtosis became large when the mutation rate (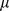) multiplied by the number of generations 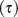 to the coalescent, then divided by twice the allele repeat length 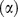 minus the allele minimum length 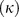 squared, was large relative to one 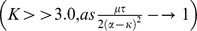. A large kurtosis also signalled that there was significant probability that the locus has undergone “microsatellite death,” or loss of its mutational activity [25]. For diploid microsatellite data comparing African vs. non-African human populations first analyzed in [15], a kurtosis of 3.02 was calculated by [25], nearly identical to the expected kurtosis under the S-SMM. In comparison, the human-chimpanzee split exhibited a kurtosis of 3.93. It was reasonable to assume, therefore, that a kurtosis very near 3.0 was the correct expectation for an individual STR allele distribution adhering very closely to the S-SMM.

Rather than attempting to calibrate the G-SMM to account for potential bias in some loci, a more practical approach to unbiased calculation of the coalescent may be to develop a method for identifying *a priori* those loci that conform most closely to expectations for the S-SMM. Using this approach, any potential bias is minimized by choosing, from available ssSTRs, those that exhibit the least deviation from the S-SMM in their observed allele distributions. We recall that the kurtosis of the idealized S-SMM is expected to be 3.0, identical to that of the normal distribution. A well-known property of the normal probability distribution is that the mean, median and mode values are the same [26]. The arithmetic mean-geometric mean inequality (AM-GM) states that the geometric mean of a set of numbers is always less than the arithmetic mean, except when all of the numbers in the set are identical [27]. Jensen’s Inequality has been used both to prove the mean-median-mode equivalency for the normal distribution and to prove the AM-GM inequality [28], [29]. These properties suggest a hypothesis, that the arithmetic and geometric means will be identical for an infinitely large, quasi-normal Poisson distribution such as the S-SMM, but that the geometric mean will be lower for non-normally distributed data and the difference will be proportional to the degree of departure from the S-SMM by the sample data set. The hypothesis is empirically testable by using computer generated S-SMM and normal distributions to compare the arithmetic and geometric means for both.

The duration of linearity for a microsatellite locus grows at the square of the allele’s range of values [15] and the maximum range of any particular STR locus is considered to be the most important constraint on the linearity of the stepwise mutation model by [20]. High kurtosis (>>3.0) signals an increased probability of microsatellite death and excessive skewness identifies those loci departing substantially from the expected allele distribution under the S-SMM. Incorporating all three of these factors into one estimator allows for a measure that can be used to identify the best available loci for improved accuracy in the calculation of 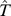 from among those available.

The present study considered the kurtosis and skewness of the probability distribution for alleles at each locus in relationship with the range of the allele values. The formula developed for this purpose was:
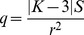
(1)where 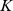 stood for kurtosis, 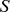 for skewness and 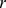 for range (highest minus lowest allele value), with the product 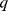 (“quality”) equaling zero for a set of allele values within an idealized locus that conformed perfectly to the S-SMM. The new expression permitted the effects of kurtosis, skewness and range of any STR locus, for which these parameters can be estimated with reasonable accuracy, to be assessed using only the observable distribution of its allele values. Because any locus that conformed very closely to the S-SMM’s expectations for kurtosis and skewness had a numerator value near zero under equation (1), while a larger range value 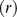 caused the denominator to grow exponentially, the limit of ratios for which the numerator remained small compared to the denominator (and the allele distribution’s kurtosis remains near 3.0) was extended either by better adherence to the S-SMM or a larger range of possible allele values.

The distribution of variances for Y-STR loci is not normal but rather log-normal [14]. The geometric mean therefore may be a better choice for calculating the average between-locus variance than the arithmetic mean. The geometric mean is analogous to the median, as it measures the median tendency of random fluctuations in probabilities across populations for factors such as random selection of gametes, birth and death rates, mutation and sampling error [30], with the median itself minimizing the sum of the absolute deviations about any point [31]. As there is substantial randomness (stochasticity) contributing to the variance of observed ASD (oASD) [14], the geometric mean may approximate the true value of the average between-locus variance better than the arithmetic mean. An approach to mitigating the problem of inaccurate estimates for 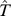 therefore is suggested. The use of the geometric mean to calculate oASD across loci and also the average mutation rate across loci 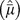 may result in 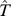 being less sensitive to errors in 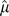, to any deviations from the S-SMM by individual loci or any variation in oASD due to sampling error or stochasticity.

In general, the current study sought to improve the accuracy of the coalescent age estimate by reducing variation caused by cryptic errors in mutation rate estimates and the use of loci that did not conform adequately to the S-SMM. The subsequent analysis identified specific procedural adjustments that were successful in reducing the amount of unexplained variation by a substantial amount, thus improving the accuracy of the 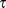 estimator.

## Materials and Methods

A comparison was made between the arithmetic and geometric means for a normal distribution by using a data set with 1.0E+8 data points, randomly generated by the “rnorm” function in the R statistical software package (http://www.r-project.org). Within the simulated normal distribution, true means of 8, 10, 20, 50, 100, 500 and 1000 were compared to calculated arithmetic and geometric means from the resulting normally distributed, randomly generated data sets. Next, the expectation that the arithmetic and geometric means were identical for an ideal S-SMM distribution was tested empirically by comparing the means from a large simulated Y-STR data set generated using the program SIMCOAL 2.0 (http://cmpg.unibe.ch/software/simcoal2/). The simulated data were intended to reflect the mutational behavior of a fully linked set of 32 loci on the Y chromosome, under the idealized S-SMM, equivalent to approximately 500,000 years of descent (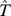 = 18,738) from an initial breeding population of 100 males.

Two sets of real population sample data, containing Y-STRs used frequently in population and forensic studies, were employed to identify loci that conformed most closely to the S-SMM. The first was a set of compiled STR allele repeat values extracted from the public online database SMGF (www.smgf.org), containing the number of times a given allele repeat value for a particular locus appeared in their Y chromosome database of approximately 35,600 haplotypes. Of the 48 STR loci available from the SMGF database, only the ssSTRs (32 loci) were used for analysis, following the recommendations of [22]. Fractional allele repeats (i.e., 10.1, 12.2), null alleles (0) and alleles with more than one repeat value detected (e.g., DYS19 with 12–15 or 13–14) were excluded from analysis, but constituted a negligible fraction of the STR values examined. Allele counts for all loci used are available online from SMGF. These data were evaluated by formula (1) to measure the degree of departure from the S-SMM, using a script written in R, available upon request. The results were sorted from lowest (“best”) to highest (“worst”) value according to the calculated 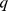 statistic for each ssSTR locus.

The second set of data was obtained from the “British Isles DNA Project,” a public database of haplotypes contributed by individuals who have been STR-tested through various commercial testing services containing 3,955 Y haplotypes with resolutions ranging from 11 to 102 STR loci per haplotype (www.familytreedna.com/public/BritishIsles). From this group, all available haplotypes containing the identical set of ssSTRs reported in the SMGF database (henceforth, STR Data Set or SDS) were extracted (n = 245). To assess the effect of the number of individual STR loci compared within a data set on the value of 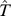, an overall estimate for the STR Data Set was compared with a series of progressively smaller subsets of the SDS. First, the 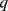 values of the 32 individual loci were calculated using equation (1), then sorted in ascending numerical order (“best to worst”). The value of 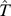 and 95% confidence intervals (CIs) for the entire set was calculated using the “Ytimeboot” program within the Matlab-based software package *Ytime* [11], with per-locus mutation rates taken from [3]. A strict stepwise mutation model (S-SMM) was selected and a starlike (‘star’) genealogy was assumed. The ancestral haplotype for the most recent common ancestor was estimated from the median allele repeat value for each locus [32]. The last locus (“worst”) in the STR data set then was removed (e.g., from 32 to 31 loci) and 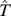 was recalculated. The process was repeated (31 to 30 loci, etc.) until only one locus remained. The resulting 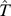
statistics and 95% CIs from each trial were plotted for comparison.

Next, the identical 32 loci from the SDS were processed in the same way, but with the per-locus 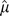 taken from [4]. Because the 32 locus data set entering the procedure each time was the same, any differences in 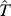
were attributable entirely to per locus differences in 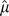 between the two studies. The 15 STR loci ranked by [20] then were subjected to the same procedure, according to the preferred order provided in Table 1 of that study, with the rates taken from [3] as in [20]. Finally, the same 15 locus subset, containing the 15 ssSTR loci maintained by YHRD, was extracted and processed in the same way but using the YHRD-maintained mutation rate averages for the loci compared in place of those from [3].

**Table 1:** Results of normal distribution simulation.

True mean	Arithmetic mean	Geometric mean	% difference
8	7.999827	7.936025	0.099695%
10	10.000038	9.949390	0.050648%
20	20.000045	19.974964	0.006270%
50	50.000153	49.990146	0.000400%
100	100.000040	99.995039	0.000050%
500	499.999940	499.998940	<0.000001%
1000	1000.000010	999.999510	<0.000001%

Difference in arithmetic and geometric means for normal distributions (n = 100,000,000). Each new calculation was made with a *de novo* normal distribution generated.

The effects of differing mutation rate estimates 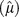 for individual loci in the STR data set on 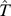
were evaluated, generating per locus estimates for 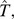 ASD and the 95% CIs for each of the three mutation rate sets compared. A linear regression (LR) model was employed to predict ASD from 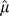 for each STR. Observed ASD was compared to 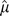 to test for a significant relationship, with individual locus values of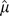 as the independent variable and ASD as the dependent variable. To test for any significant interaction between ASD and 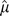, a multiple regression analysis also was conducted for each mutation rate set, using the model:
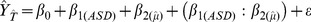
(2)


R was used for the evaluation of all linear regression models.

A hypothetical cause of differences in ssSTR mutation rates may be the minimum allele length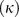 for a given locus, with shorter minimum alleles possibly corresponding to lower mutation rates. To test for this possibility, LR analyses were performed for mutation rate estimates 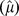 from both [3] and [4] with 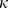 as the independent variable and 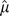 as the dependent variable. A similar LR was performed to test for a possible correlation between 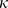 and the STR’s calculated *q* value, with 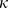 as the independent variable and *q* as the dependent variable. Both comparisons used the allele data from SMGF for the 32 locus set.

The difference between the arithmetic and geometric means for a sample’s probability distribution was exploited to measure the percentage degree of departure by a sample set of haplotypes from the S-SMM. To assess the degree of departure for a given population sample from the S-STR, 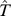 was calculated for the set of simulated haplotypes using both the geometric and arithmetic means and the difference (*D*) between them was measured using the formula:
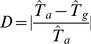
(3)where 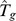 stood for the geometric average of 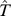
and 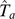 stood for the arithmetic average. *D* statistics were calculated for relevant combinations of actual STR data and mutation rates compared in the study.

Although the central 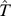
point estimate is independent of the shape of the genealogy of a population, the corresponding confidence intervals are strongly dependent on the (often unknown) growth rate of the sampled population. Except for the case of an extreme population bottleneck, though, the actual CIs will be bounded by the ones calculated for zero growth rate (a constant effective population size) and an exponentially growing population in which all lines survive to the present and are represented in the sample (a “starlike” genealogy) [17]. Using *Ytime*, the effects of different population genealogies on both the central 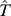
statistic and the resulting CIs were investigated. The *Ytime* parameter *Rgrowth* defined a scaled growth rate for the population sample being analyzed, with the parameter specified by 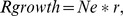 where
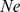
was the current effective population size and 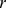 was the instantaneous growth rate per generation. A no-growth, constant-sized population was specified in *Ytime* by using 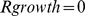; any intermediate parameter value from zero to ‘star’ (quasi-infinite exponential) growth was available. The *Rgrowth* parameter was varied between zero and ‘star’ by eleven orders of magnitude (from 0.0E+0 to 1.0E+11) and the effects on 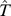 and the 95% CIs were compared for each growth rate, using the SDS with 32 loci.

## Results

Comparing the means of several sets of normal distributions (1.0E+8) data points randomly generated for each set, it was determined that the arithmetic and geometric means deviated by a maximum of 0.1% with a true mean value of 8 and with the difference diminishing to less than a millionth of a percent with a true mean value of 500 and 1000 (Table 1). For the idealized S-SMM simulated data set, the ratio of the difference between the arithmetic mean and the geometric mean for 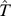 was calculated to be 0.06%, with a kurtosis of 3.027 and skewness of −0.002, conforming closely to expectations for the S-SMM as described by [24]. These results supported the inference that an infinitely large data set conforming perfectly to the S-SMM would have identical geometric and arithmetic means, and that any difference in the means reflected the degree of divergence from the ideal S-SMM for the sample data set.

**Figure 1:**
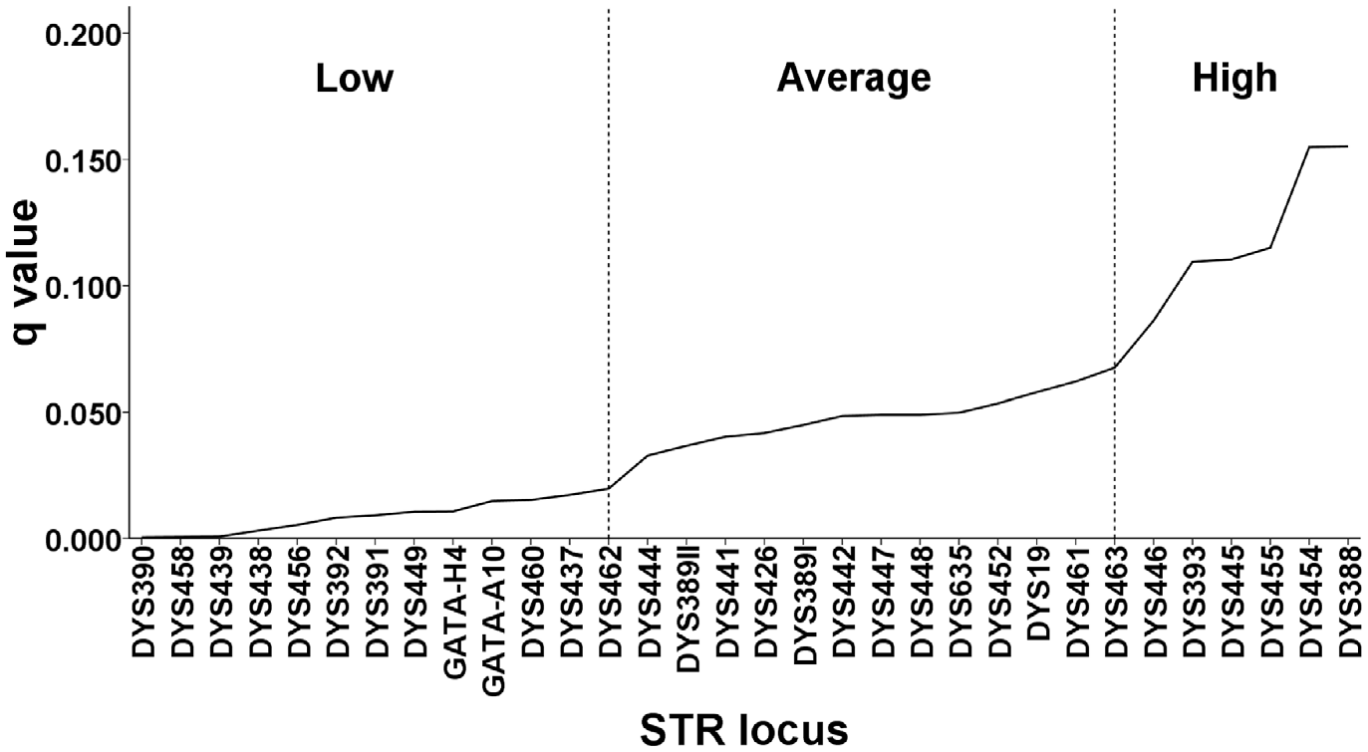
Calculated *q* values for 32 loci, SMGF data set. A lower *q* value equals a better fit to the S-SMM for an individual locus.

Using equation (1), the lowest calculated 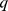 values were used to identify loci that departed the least from the S-SMM and therefore were preferred for the purpose of calculating 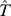 (Table 2). In graphing the relative values of the 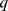 statistic for the test loci, two noticeable changes in the slope of the curve occur; the first after DYS 462 and the second after DYS635. The resulting values for 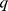 thus fell into easily identifiable ranges of “best”, “average” and “worst” conformity to the S-SMM, for these 32 loci (Figure 1).

**Table 2:** 32 commonly used, single-site STRs and their q values.

STR locus	*q* value	STR locus	*q* value
*DYS390*	0.000412	*DYS426*	0.041725
*DYS458*	0.000603	*DYS389I*	0.044951
*DYS439*	0.000744	*DYS442*	0.048485
*DYS438*	0.003111	*DYS447*	0.048934
*DYS456*	0.005350	*DYS448*	0.048934
*DYS392*	0.008238	*DYS635*	0.049764
*DYS391*	0.009169	*DYS452*	0.053431
*DYS449*	0.010557	*DYS19*	0.057823
*GATA-H4*	0.010700	*DYS461*	0.062045
*GATA-A10*	0.014808	*DYS463*	0.067503
*DYS460*	0.015247	*DYS446*	0.086166
*DYS437*	0.017258	*DYS393*	0.109470
*DYS462*	0.019705	*DYS445*	0.110377
*DYS444*	0.032796	*DYS455*	0.115019
*DYS389II*	0.036689	*DYS454*	0.154864
*DYS441*	0.040277	*DYS388*	0.155135

Loci are ranked in order of conformity to the strict stepwise mutation model (S-SMM) using equation (1). The lowest 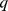 value identifies the best fit to the S-SMM, using the SMGF summary statistics for 32 loci (n≈35,600).

**Figure 2:**
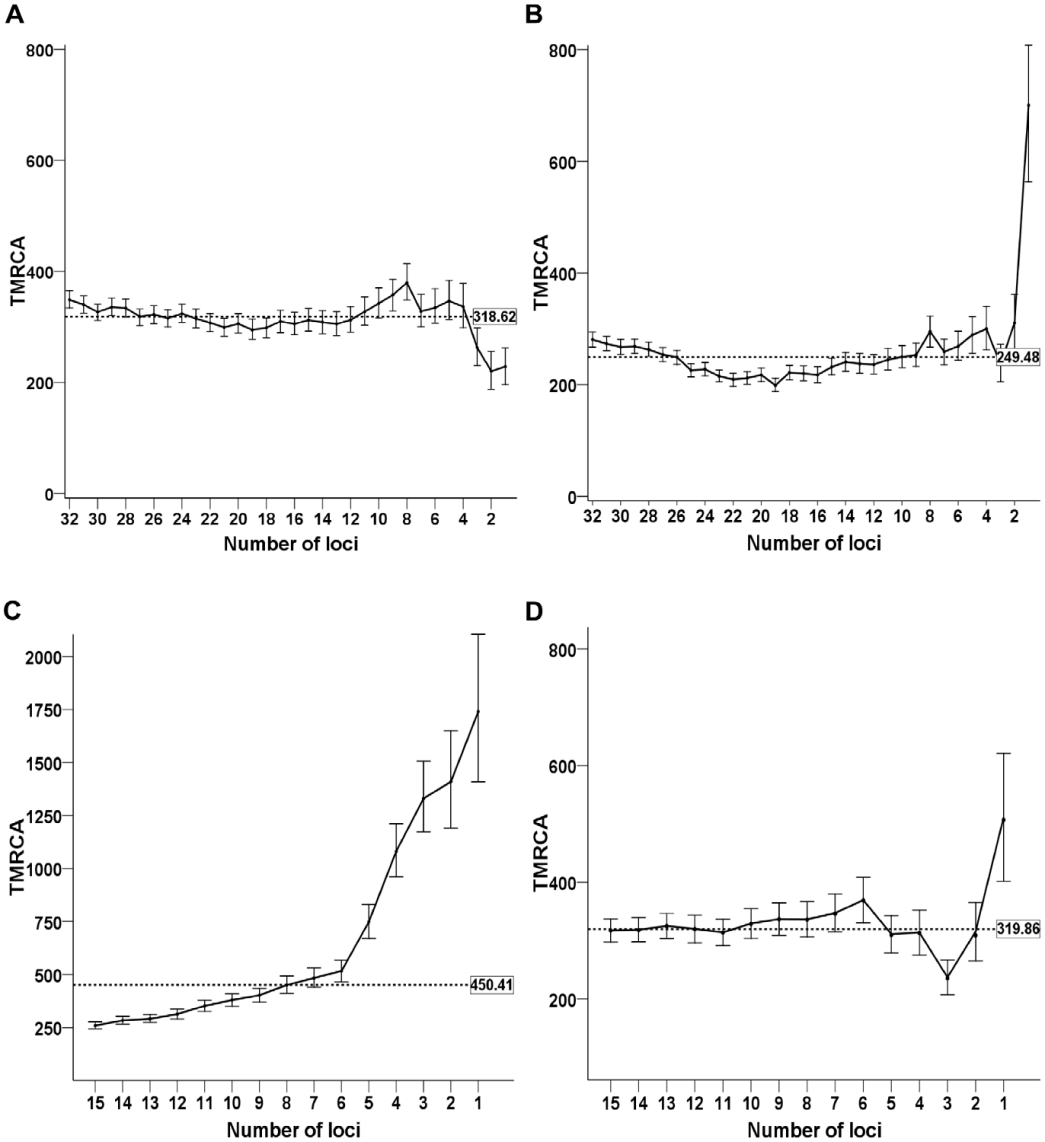
Coalescent calculations (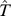) and confidence intervals. A) Burgarella mutation rates, 32 loci, best to worst order. B) Ballantyne mutation rates, 32 loci, best to worst order. C) Busby et al., 2012, 15 recommended loci, best to worst order. D) YHRD mutation rates, best to worst order, 15 loci. TMRCA is given in generations. Error bars represent 95% confidence intervals for each TMRCA estimate. Dashed lines indicate the median 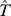 value for each comparison, shown in the box at the right end of the dashed line.

Estimates of 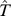 based on diminishing numbers of loci extracted from the same data set, in which those loci with greater deviations from the S-SMM were removed first, varied from 220 generations to 380, a difference of 72% between the lowest and highest value when using the mutation rates from [4] (Figure 2A). When the mutation rates from [3] were substituted, the range from the lowest to highest 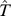 varied from 281 generations to 701 generations, a difference of 249% (Figure 2B). Similar results were obtained for the 15 locus data sets (Figure 2C, 2D.) The stability and linearity of 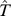 deteriorated with decreasing numbers of loci in all cases.

**Figure 3:**
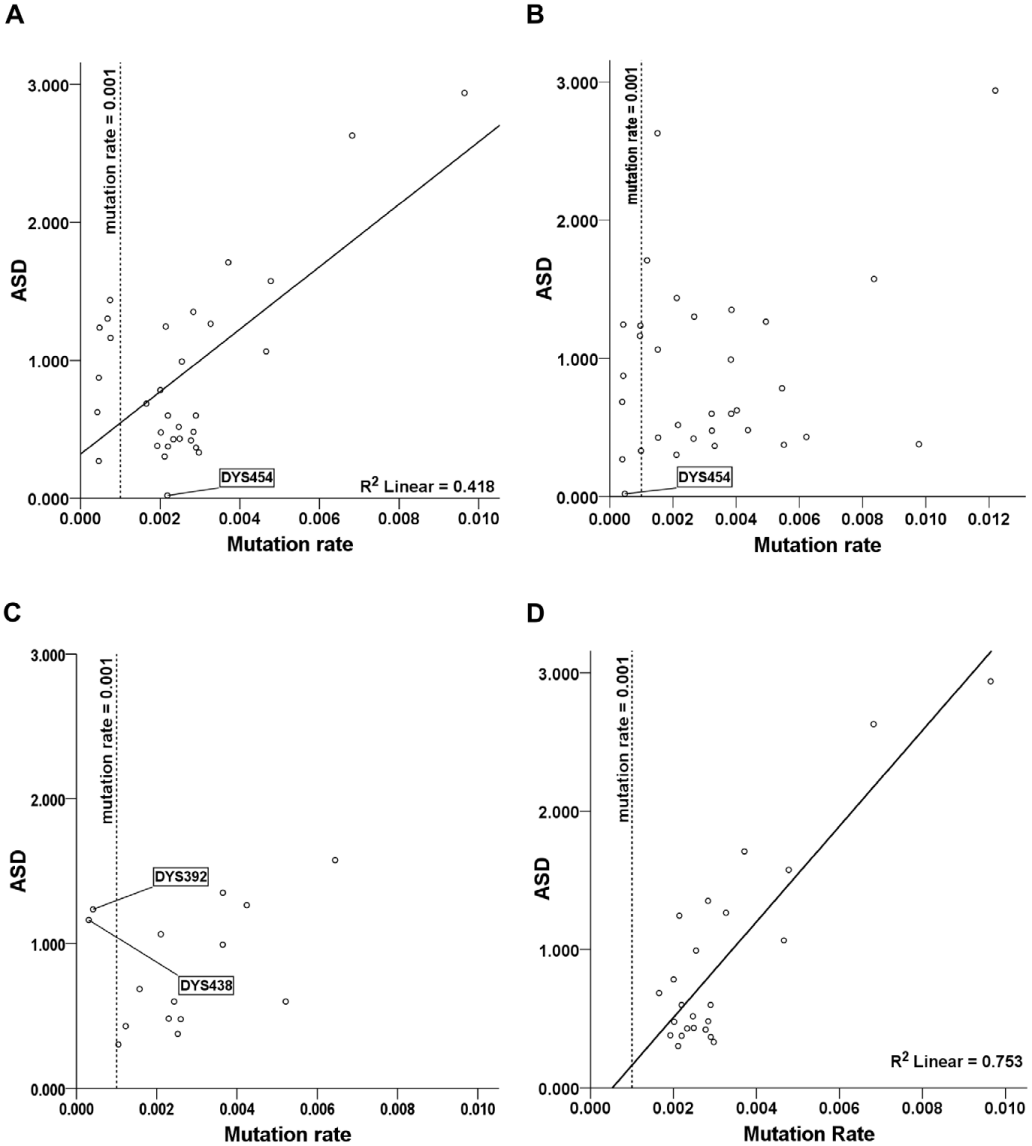
Regression coefficients and confidence intervals. ASD for each locus compared with mutation rates for each locus, using three published studies. A) Burgarella rates, 32 loci. Loci identified in bold face (e.g., DYS448) fall within the 95% mean confidence interval for the locus set compared. B) Ballantyne rates, 32 loci. C) YHRD, 15 loci, YHRD rates. D) Burgarella rates, 24 loci, with loci that have 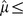
0.001 removed from the calculation. Data set compared is from the “British Isles DNA Project,” n = 245, 32 locus haplotypes and subsets of the same data set (24 locus reduced set and 15 locus set corresponding to the YHRD ssSTR loci.).

Mutation rates from [4] showed a significant relationship between 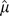 and ASD (t = 4.645, df = 30, p<0.0001, R^2^ = 0.4184) but with a large portion of the variation (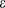) remaining unexplained (Figure 3A). For the estimates from [3], ASD was not significantly related to 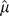 (t = 1.555, df = 30, p = 0.1304, R^2^ = 0.0746), an unexpected result (Figure 3B). The YHRD 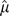 rates (Figure 3C) also were not significantly related to ASD (t = 1.389, df = 30, p = 0.1880, R^2^ = 0.1293). When data for the seven loci with 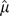
<0.001 were removed from the LR data, however, the amount of explained variation in ASD due to 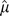 (rates from [4]) increased sharply (Figure 3D), with R^2^ = 0.7535 (t = 8.20, df = 22, p<0.0001). Subjecting rates from [3] to the removal of all loci with 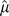
<0.001 did not improve the non-significant relationship between ASD and 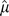 (t = 1.147, df = 22, p = 0.2636, R^2^ = 0.056). Removing two loci from the 15 YHRD loci with 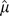
<0.001 (DYS438 and DYS392) and also DYS389I (collinear with DYS389II) improved the correlation coefficient between 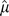 and ASD for the remaining 12 loci to a statistically significant level (R^2^ = 0.4921, t = 3.199, df = 10, p = 0.01101).

With the mutation rate estimates from [4], there was a significant relationship detected between ASD and the response variable 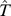 (t = −6.480, df = 28, p<0.0001) and between 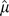 and 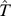 (t = 4.109, df = 28, p = 0.0003), but not for the interaction 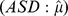 (t = 1.748, df = 28, p = 0.0914). With estimates from [3], there was a significant relationship between ASD and 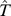 (t = 3.682, df = 28, p = 0.0010) and between 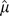 and 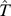 (t = −2.423, df = 28, p = 0.0221) but the interaction 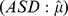 again was not significant (t = −0.937, df = 28, p = 0.3570). The 15 locus YHRD mutation rate set showed a significant relationship between ASD and 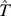 (t = 3.598, df = 11, p = 0.0042), but this time the relationship between 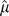 and 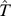 was not significant (t = −0.302, df = 11, p = 0.7683) with 15 loci. However, when DYS438, DYS392 and DYS389I were removed from the allele set, the relationship between 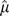 and 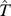 became significant (t = −3.192, df = 8, p = 0.01276). The interaction 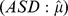 remained non-significant (t = −0.583, df = 8, p = 0.57616) for the reduced data set.

Linear regression analyses comparing the minimum allele length 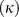 for a given locus with its estimated mutation rate 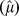 found no significant correlation between 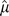 and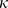 using rate estimate from [3] (t = 1.275, df = 30, p = 0.212) and [4] (t = 0.061, df = 30, p = 0.952). A comparison of 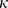 for a given locus with its calculated *q* value (which is not dependent on 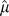) also found that the correlation was not significant (t = −1.027, df = 30, p = 0.313).

The calculation of *D* for several sets of loci and mutation rate combinations determined that the closest agreement between the two averages was calculated by using mutation rates from [4] with the full 32 locus set (Table 3). The overall geometric mean of the 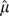 rates for the 32 loci from [4] was 0.0020 per generation, and for the rates from [3] 0.0021, a 5% difference, compared to a mean of 0.0026 for [4] and 0.0032 for [3], a 23% difference for the arithmetic average.

**Table 3:** Comparison of arithmetic and geometric means for 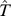.

Mutation rates	No. of loci	Geometric mean		Arithmetic mean		*D* (% difference)	
Burgarella	32		337.8274	348.7863	3.1%		
Ballantyne	15		294.3013	259.0993	13.6%		
Ballantyne	32		321.066	280.7067	14.4%		
Burgarella	15		360.613	314.5164	14.7%		
YHRD	15		367.785	317.3084	15.9%		

Difference (*D*) from equation (2) is a measure of the overall conformity of the data to the distribution expected under S-SMM. The lowest percentage difference indicates the least amount of departure from the S-SMM.

**Figure 4:**
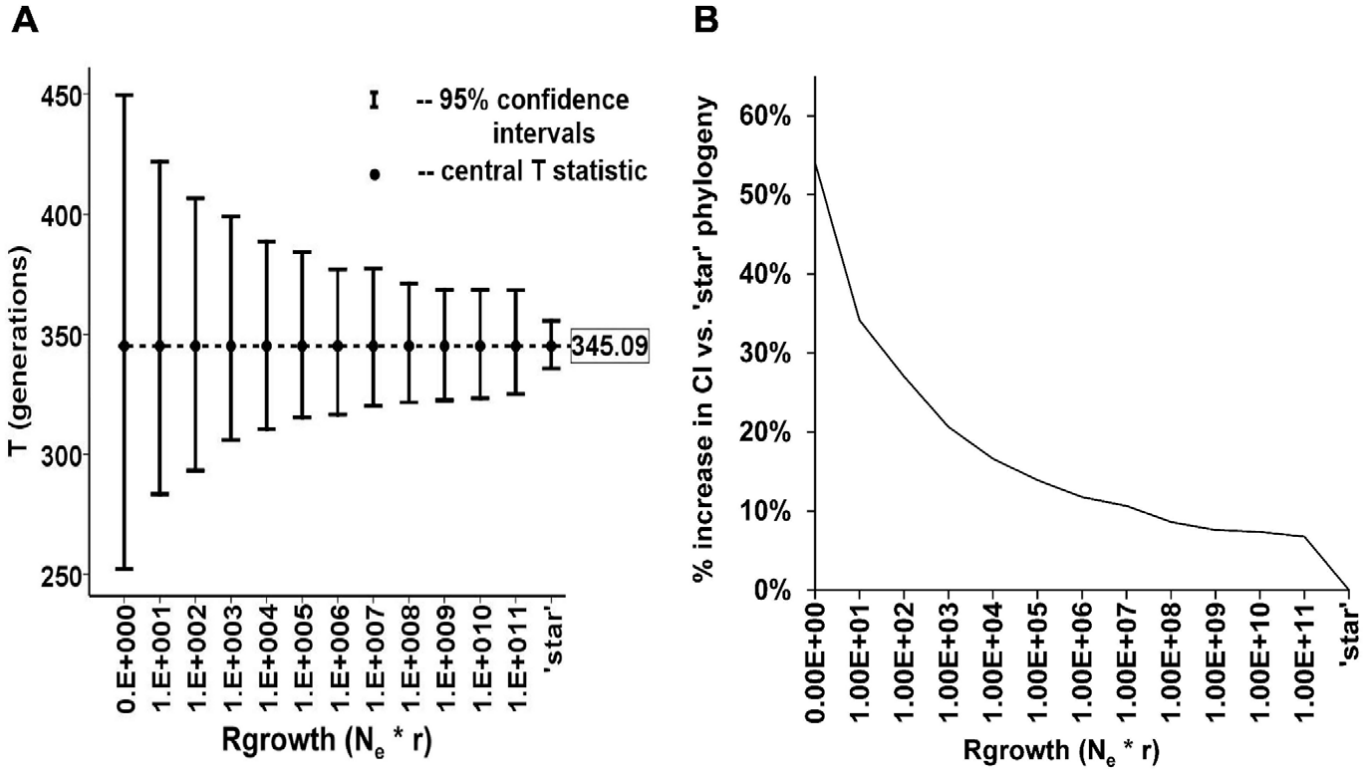
32 locus STR data set with changing growth rates and genealogy shapes. A) 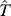
and 95% confidence intervals plotted for the STR data set (n = 245, 32 loci) as the *Rgrowth* (N_e_*r) parameter is varied between *Rgrowth* = 0 (no growth, constant sized population) to a “star” genealogy (approximating infinite exponential growth by all descendant lines) by orders of magnitude from 1.0E+1 to 1.0E+11. 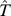 = 345.09 was the median value for all *Rgrowth* models and was shown empirically to be independent of the shape of the sampled genealogy, growth rate and population size. B) Percentage change of 95% CI overestimation compared to ‘star’ genealogy for the full range of comparisons in 4A.

To test for the effect of different growth model selections on 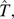 *Ytime* was run under several growth rate scenarios, varying by orders of magnitude from zero growth to ‘star’ for the STR data set (n = 235). Substitution of the no-growth model (*Rgrowth* = 0) for the ‘star’ genealogy model produced no change in the point estimate 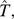 but did increase the width of the 95% confidence intervals substantially, by approximately 54% for the no-growth model compared to the star genealogy. For any value of *Rgrowth* at or above 1000, the average increase in 95% confidence intervals over the star genealogy was 21% or less, approaching a limit of about 7% above 1.0E+10 (Figure 4).

## Discussion

A fundamental question for any Y-STR based research study or application is: how many STR loci are needed and which of the available loci will be the most useful for the purpose at hand? The purpose of this study was to provide an exploratory framework for the analysis and selection of the most suitable currently available Y-STRs for calculation of the coalescent and for the future evaluation of those STRs on the Y chromosome yet to be identified. In the process, two statistics for quantifying the degree of departure by any Y-STR from the S-SMM were developed, with *q* (equation 1) dependent only upon the availability of empirical data concerning allele repeat values and frequencies and *D* (equation 3), a normalized statistic independent of any parameter save the need to provide a reasonable estimate of the average mutation rate 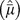 across all loci compared.

In principle, the 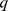 statistic developed here can be used effectively with any STR to evaluate its suitability for the purpose of calculating 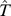 and, given a large enough allele data sample, to allow an evaluation of its behavior with regard to the S-SMM. Equation (1) may be incorporated easily into a spreadsheet with the caveat that the method of calculating kurtosis used by many spreadsheets returns an “excess” kurtosis value (kurtosis in excess of 3.0), therefore the parameter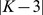 (kurtosis minus 3.0) from Equation (1) becomes 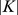 (kurtosis in excess of 3.0).

To illustrate the procedure, 93 ssSTRs routinely available from commercial testing companies were evaluated using those haplotypes from the SDS that were tested for all 93 loci (n = 235) in *Excel*, with the results ranked from best to worst in Supplementary Table S1 (ST1). Differences in rank order between Table 2 and ST1 were attributable to sample size and sampling error, with the SMGF sample far exceeding the SDS (n≈35,600 vs. n = 235), resulting in some variation in allele distribution at any given locus compared between samples. Because of these differences, the results in Table 2 were regarded as more robust. Some loci found in ST1 clearly do not provide an accurate estimate of 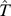 due to excessive kurtosis, skewness, a narrow range, or a combination of all three (e.g., DYS472). Inclusion of loci that conform poorly to the SMM in coalescent calculations will result in inaccurate estimates of 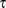. Considering the results from the two data sets and Figure 1 as a whole, a “cutoff” value of *q* = 0.07 is suggested, permitting only loci that have good to excellent conformity to the S-SMM to be used when estimating 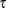.

ASD is not dependent on population size or the specific shape of the genealogy [16], [17] and because 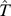
is dependent only upon ASD and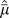 for its calculation, it too is independent of population size and genealogy shape. By examining different growth models, from a no-growth, constant size population to one with infinite growth, it was confirmed empirically (Figure 4) that the 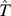
estimate was unaffected by the specific genealogy of the sample but that confidence intervals were affected, with the no-growth, constant-size model having the most conservative (widest) CIs. Therefore, any comparison of the arithmetic and geometric means (equation 3) will be independent of population size or genealogical history as only the point estimates 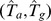 are used in calculating *D*.

A basic limitation in calculating the coalescent from ssSTRs is that 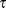
cannot be extended beyond the MRCA for all members of a given haplogroup. Each Y haplogroup must coalesce no earlier than the first individual male who acquired the defining Y-SNP [9]. It is possible that the MRCA for a haplogroup currently represented in a living population was not the first person to acquire the SNP, but merely the most recent common ancestor of those particular Y lines that have survived to the present, with other lines from the same founding haplotype having gone extinct. Thus, although ASD is an unbiased estimator of the TMRCA for a specific haplogroup for any length of time up to at least 2 million years before present [16], it cannot always provide an estimate of the actual founding time of the haplogroup.

At the opposite end of the time scale a very recent founding event, with the defining Y-SNP having emerged only in the past several generations, may result in a population that has not yet have acquired sufficient genetic diversity among descendants of the founder to calculate 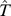 accurately, because STR mutation is a stochastic process and 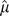 is only the central estimate of the average mutation rate over time. This limitation is especially important for samples that have few ssSTR loci to compare. The largest differences between adjacent 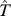 values, as the number of loci was reduced, occurred when there were fewer than six loci compared (Figure 2) and suggested that a minimum of six ssSTR loci with good conformity to the S-SMM were needed when calculating 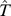 to avoid wholly unreliable estimates. More than six loci should be used whenever possible, regardless of time depth, to further improve the accuracy of 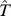
as long as the additional ssSTRs also conform closely to the S-SMM. The more loci compared, the narrower the confidence intervals will become, improving precision as well as accuracy. The use of many ssSTRs per haplotype reduces the problem of shallow TMRCAs because there is a higher cumulative probability that at least some loci have undergone mutation within the past few generations. Even after dropping DYS389I/II, DYS438 and DYS392 from the calculation of 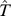, there were still 45 ssSTRs remaining in Supplementary Table S1 with *q* <0.07, a sufficiently high number of loci for most purposes and greatly exceeding the resolution of the 16 “standard” YHRD loci used by many studies presently.

The size of errors associated with 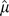 depended partly upon the range of the mutation rate, with higher rates having smaller errors [4]. No significant relationship was found, however, between the minimum allele length 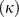 and 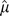 for the 32 Y STR loci from SMGF for either [3] or [4] or between the minimum allele length 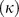 for a given locus and its calculated *q* value. Furthermore, the failure of 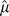 to correlate significantly with ASD for two of the three sets of mutation rate estimates (Figure 3) suggested that some mutation rate estimates introduced substantial, uncorrected errors to the calculation of 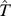, as the allele values (and the resulting oASD statistics) entering each calculation were the same for the particular locus being compared and only 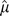 was varied.

The sharp increase in the proportion of explained variability between the 32 locus data set and the reduced 24 locus set (using 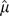 from [4] – Figure 3A,D) is notable and suggests that estimates for loci with mutation rates below 0.001 per generation may not be suitable presently for the calculation of 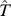, especially when there is little or no father-son direct observational evidence available for the estimate. The absence of a significant interaction between 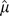 and ASD for any of the comparisons made, though, suggests that the relationship between these two factors is not dependent upon any absolute value of 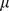, but rather is independent of the rates’ ranges. Put differently, a low, medium or high 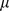 rate is not likely to be the cause for 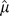
’s failure to predict ASD, but rather that the estimation errors for low rates are greater than for higher rates due to fewer available direct observations from which to estimate the “true” rate for 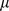. If the true 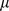 for all loci compared actually were available, any remaining errors in the 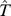
estimate would be reduced to those introduced by stochasticity, genetic drift and sampling error. By removing those loci for which 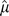
<0.001 from the calculation, the amount of unexplained variance due to factors such as error in estimates of 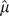, sampling error, unequal numbers of offspring and stochasticity in the mutation process was reduced to 24.7% for the 24 locus subset (Figure 3D, with 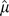 estimates from [4]), a substantial improvement over the first comparison (Figure 3A), where 58.2% of the total variation was left unexplained.

Linear regression analyses of YHRD loci with and without DYS392, DYS438 and DYS389I highlighted significant bias in 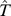
when these loci first were included (Figure 3C) and then removed (Figure 3D). Their removal from the YHRD loci strengthened correlation from a non-significant result to a significant one (R^2^ = 0.4921, with mutation rates from [3]), suggesting that estimates based on the 12 selected YHRD ssSTR loci are reasonably reliable if no higher resolution data is available. Using mutation rate estimates from [4] with the YHRD loci, the correlation coefficient for the 12 selected YHRD loci improved slightly more, with R^2^ = 0.5027. Because many studies have been published with only the standard YHRD loci available in the data set, the improvements described here are directly applicable to the calculation of 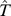 from these data. Further improvements seen here in the accuracy of 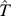 resulted directly from an increase in the number of loci from 12 to 24 (from 50.27% to 75.35% of variation in ASD explained by 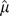, mutation rate estimates from [4]), strongly supporting the need for increased numbers of ssSTR loci when planning research projects that include a calculation of the coalescent.

Given the significant relationship detected between 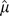 and 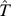, it was reasonable to infer that the specific choice of 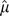 for each locus had the strongest effect on the calculated value of 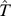. Because 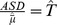, any upward bias in 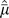 introduced by use of the arithmetic mean when averaging mutation rate estimates across loci had the effect of biasing 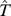 downward. Use of the geometric mean provided a more consistent measure of 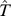 when using different sets of loci because it returned the median value for the average of the observed per-locus variances (oASD) and for the individual 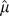 values rather than a potentially skewed 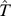 based on the arithmetic means for one or both variables.

While using the geometric mean to determine the average within-locus observed variance across loci (oASD), precautions were taken to remove any ssSTRs from the sample data set that had zero variance (i.e., all haplotypes have the same allele value as the ancestral haplotype at the locus of interest) because normal computer-based methods of calculating the geometric mean would have resulted in an undefined result for log(0). The issue of zero allelic variance within a specific locus is more likely to occur within data sets that have only a few haplotypes or loci being compared. Within a larger sample, any locus exhibiting zero variance between the sample and the ancestral (median) allele value is fundamentally uninformative with regard to the calculation of 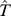 and may be deviating substantially from the S-SMM. These uninformative loci, lacking in variation, should be dropped from calculations regardless (even while using the arithmetic mean) because a substantial downward bias of the average between-locus variance may be introduced by them, resulting in an underestimation of 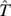. For example, it was clear from Figure 3A and 3B that DYS454 did not appear to be accumulating any appreciable amount of variance, especially given its relatively fast mutation rate estimate (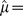 0.002182 by [4]). It was suggested by the empirical data that this locus may be undergoing microsatellite death. Regardless of the cause, DYS454 was uninformative with regard to the coalescent in this context and its removal from STR haplotype data sets was necessary to avoid a downward bias in 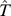.

Though there is no reason to believe that mutational behavior for ssSTRs will differ between male Y haplogroups or geographic regions, it should be noted that both population samples used here (SMGF, SDS) contain Y haplotypes dominated by male lineages originating in Europe and may not be fully representative of ssSTR allele distributions in other regions of the world. Some Y-STR databases, such as SMGF, have many loci per haplotype available and contain many haplotypes, but are biased toward descendants of a particular ancestral population (European), due to their sample collection strategies. Others, such as YHRD, have excellent coverage of many geographic regions but relatively few loci available. Because of this, *q* values developed using the SMGF and SDS data regarding the suitability of a particular Y STR locus for estimation of 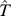
using the S-SMM and reported in Table 2 and ST1 should be viewed as preliminary, though the very large sample size for the SMGF data set is likely to compensate for any unknown bias introduced by incomplete geographic coverage. Further research, including a worldwide survey of Y STR allele frequencies at each of the loci described in Table 2 and in ST1, will help to further refine the results presented here concerning the conformity of a given ssSTR locus to the S-SMM.

Even within the geographic limitations of the available test data, though, the use of equations (1) and (3) in choosing and evaluating STRs lead to substantially improved estimates of 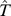. When combined with the use of the geometric mean in place of the arithmetic mean for averaging both ASD and 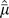, the overall percentage of unexplained error for 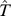 becomes mainly dependent on sample size, variations in numbers of offspring, and the inherent stochasticity of the mutation and gamete selection processes, leading to more robust estimates of the coalescent 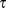 with less unexplained error. Having taken into account factors of skew, kurtosis, allele value ranges and having identified those loci that are likely to contribute bias when calculating the coalescent, it also is evident from the statistical analysis presented here that the accuracy of 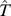 calculations will benefit most from future refinements in the estimation of ssSTR mutation rates, with the logistic regression model [4] currently providing 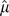 estimates that are more consistent with ASD than the Bayesian posterior distribution analysis approach [3]. The problem is mitigated, however, by the use of the geometric mean for averaging both ASD and 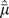 when calculating 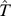 rather than the arithmetic mean.

## Supporting Information

Table S1 **List of 93 commercially available Y chromosomes ssSTRs ranked by** ***q*** **value.** Lowest *q* value equals best conformity to the S-SMM.
(XLS)
